# Life Expectancy of Transplanted Livers: HOPE Against Aging?

**DOI:** 10.1097/SLA.0000000000006883

**Published:** 2025-08-07

**Authors:** Janina Eden, Philip C. Müller, Christoph Kuemmerli, Noa Aegerter, Isabel M.A. Brüggenwirth, Gabriela Berlakovich, Bettina M. Buchholz, Florin Botea, Stefania Camagni, Matteo Cescon, Umberto Cillo, Fabio Colli, Luciano G. De Carlis, Riccardo De Carlis, Fabrizio Di Benedetto, Jule Dingfelder, Dulce Diogo, Daniele Dondossola, Moritz Drefs, Jiri Fronek, Giuliana Germinario, Enrico Gringeri, Christiano Guidetti, Georg Györi, Matej Kocik, Efrayim H. Küçükerbil, Dionysios Koliogiannis, Georg Lurje, Paolo Magistri, Diethard Monbaliu, Mostafa el Moumni, Beat Müller, Damiano Patrono, Wojciech G. Polak, Robert J. Porte, Matteo Ravaioli, Michel Rayar, Renato Romagnoli, Gustaf Sörensen, Deniz Uluk, Pierre A. Clavien, Vincent E. de Meijer, Philipp Dutkowski

**Affiliations:** * Department of Surgery, Section of HPB Surgery and Liver Transplantation, University of Groningen and University Medical Center Groningen, Groningen, The Netherlands; † UMCG Comprehensive Transplant Center, Groningen, The Netherlands; ‡ Department of Visceral Surgery, University of Basel, Clarunis, Basel, Switzerland; § Division of Transplantation, Medical University of Vienna, Vienna, Austria; ∥ Department of Visceral Transplantation, University Medical Center Hamburg-Eppendorf, Hamburg, Germany; ¶ Fundeni Clinical Institute, Center of General Surgery and Liver Transplantation, Titu Maiorescu University, Bucharest, Romania; # Department of Organ Failure and Transplantation, ASST Papa Giovanni XXIII, Bergamo, Italy; ** Department of General Surgery and Transplantation, IRCCS, Azienda Ospedaliero-Universitaria of Bologna, University of Bologna, Bologna, Italy; †† Chirurgia Generale 2, Hepato-Biliary-Pancreatic Unit and Liver Transplant Center, Padova University Hospital, Padova, Italy; ‡‡ General Surgery 2U, Liver Transplant Centre, Azienda Ospedaliero Universitaria Città della Salute e della Scienza di Torino, Turin, Italy; §§ Department of General Surgery and Transplantation, ASST Grande Ospedale Metropolitano Niguarda, Milan, Italy; ∥∥ Hepato-Pancreato-Biliary Surgery and Liver Transplantation Unit, University of Modena and Reggio Emilia, Modena, Italy; ¶¶ Adult Liver Transplantation Unit, Department of Surgery and Gastroenterology, Coimbra Hospital and University Center, Coimbra, Portugal; ## General and Liver Transplant Surgery Unit, Fondazione IRCCS Ca’ Granda Ospedale Maggiore Policlinico, and Department of Pathophysiology and Transplantation Università degli Studi di Milano, Milan, Italy; *** Department of Transplant Surgery, University of Munich Grosshadern, Munich, Germany; ††† Transplant Surgery Department, Institute for Clinical and Experimental Medicine (IKEM), Prague, Czech Republic; ‡‡‡ Erasmus MC Transplant Institute, University Medical Center Rotterdam, Division of HPB and Transplant Surgery, Rotterdam, The Netherlands; §§§ Department of Surgery, Universitätsklinikum Heidelberg, Heidelberg, Germany; ∥∥∥ Department of Abdominal Transplantation, Leuven Transplant Center, University Hospitals Leuven, Leuven, Belgium; ¶¶¶ Department of Surgery, Section of Epidemiology and Statistics, University of Groningen and University Medical Center Groningen, Groningen, The Netherlands; ### CHU Rennes, Service de Chirurgie Hépatobiliaire et Digestive, Rennes, France; **** Transplant Institute, Sahlgrenska University Hospital, Gothenburg, Sweden; †††† Swiss HPB and Transplant Center, Department of Visceral Surgery and Transplantation, University Hospital Zurich, Zurich, Switzerland

**Keywords:** cumulative liver age, liver transplantation, machine liver perfusion

## Abstract

**Objective:**

To investigate the impact of cumulative liver age on graft survival in liver transplant recipients.

**Background:**

Organ shortage has led to increased use of elderly donor livers for an ageing group of recipients, particularly in Europe. We hypothesized that hypothermic oxygenated perfusion (HOPE) enhances graft resilience against aging in livers from elderly donors.

**Methods:**

This post hoc analysis of the multicenter European HOPE-REAL study (NCT05520320) examined liver age in adult recipients of HOPE-treated brain-dead donor (DBD) livers. Cumulative liver age was defined as the sum of donor age and post-transplant graft survival. HOPE-treated DBD liver transplants were compared with a single-center control cohort of non-perfused DBD livers using propensity score matching to adjust for confounders.

**Results:**

The median cumulative liver age was significantly higher in the HOPE-treated (n=768) cohort than in the non-perfused (n=863) group (69 vs 61 years, *P*<0.001), yet graft survival was superior in the HOPE-treated group (log-rank *P*<0.001). After matching, cumulative liver age remained significantly higher in the HOPE group (*P*<0.001). Multivariate analysis identified HOPE as an independent predictor of cumulative liver age (*P*<0.001).

**Conclusions:**

HOPE treatment seems to mitigate the risks associated with transplanting elderly DBD livers, leading to excellent long-term survival in an ageing recipient population. These findings support broader adoption of HOPE to improve utilization of older donor livers and expand the donor pool.

Aging is a fundamental biological process characterized by a general decline in organ function and an increased risk of disease. Key molecular mechanisms involved include the adenosine monophosphate-activated kinase (AMPK), Sirtuin 1 (SIRT1), and mammalian target of rapamycin (mTOR) pathways.^1^ Although aging is generally considered irreversible, it may be delayed by stimulating longevity pathways or inhibiting aging-related processes. Despite these possibilities, advanced donor age has traditionally been considered a decisive risk factor for graft and patient survival after liver transplantation and most donor risk scores therefore include donor age as an important confounder for outcome.^2–4^ However, organ shortage led to increased use of liver grafts from elderly donors, even for an ageing group of recipients, particularly in Europe.^5,6^ Accordingly, the median donor age is 56 years within the Euro-Transplant region, compared with 43 years in the US region. Nevertheless, excellent long-term outcomes have been reported in European liver transplant recipients,^7^ most recently for example also after transplantation of elderly high risk DCD livers treated by hypothermic oxygenated perfusion (HOPE).^8^

Machine perfusion approaches have recently been advocated to improve organ quality,^9^ but no data exist on the actual cumulative liver age of machine-perfused transplanted livers. Given the recently described mitochondrial repair effects of HOPE,^10^ we hypothesized that machine perfusion, specifically HOPE, may enhance resistance against aging in livers from elderly donors when compared with a historical cohort of unperfused livers. We analyzed for this purpose a recent large cohort of HOPE-treated DBD livers and compared cumulative liver age, summarizing donor age and recipient follow-up, with an unperfused cohort from a singly center. The data suggest that HOPE-treated and transplanted livers may have an extraordinary liver life span.

## METHODS

### Study Design

This is a post hoc analysis of the multicenter European HOPE-REAL observational study (NCT05520320) on liver transplantation after machine perfusion using HOPE.^8^ The HOPE-REAL cohort originally includes adult recipients from 22 European liver transplant centers who received a DBD or DCD liver grafts treated with HOPE, dual-HOPE (DHOPE), or NRP HOPE between 2012 and 2022. Eligibility criteria for participating centers have been detailed previously.^8^ In summary, centers were eligible after performing at least 20 HOPE procedures, and all consecutive HOPE cases outside clinical trials were included in this analysis. Data collection extended until December 31, 2022, ensuring a minimum follow-up time of 12 months for each patient.

### Data Collection

Data collection and analysis were approved by the local institutional review boards of Zurich (KEK No. 2019-01000) and Groningen (No. 2021-00366). Donor variables included age, biological sex (female, male), donor height, donor BMI, donor cause of death, graft type (DBD or DCD), total warm ischemia time (time interval between donor withdrawal of life support and cold flush), functional warm ischemia time (time interval between blood pressure <50 mmHg and start of cold in situ flush), and asystolic warm ischemia time (time interval between cardiac arrest and cold in situ flush). Preservation parameters consisted of cold ischemia time (time interval between cold in situ aortic flush and start of cold perfusion), perfusion time, perfusion duration, perfusion flow, perfusion type (HOPE or dual-HOPE), and the device type (VitaSmart, Bridge2Life, Wandsworth, England; Liver- Assist, XVIVO, Groningen, the Netherlands; other devices). Recipient parameters included age and biological sex (female, male), laboratory MELD score, the balance of risk score, primary transplants or retransplants, the presence of hepatocellular carcinoma (HCC), tumor size, tumor number, and alpha fetoprotein.

### Outcome Parameters

For the current study, we calculated the cumulative liver age of the DBD liver transplant cohort transplanted between 2015 and 2022. Cumulative liver age was defined as the sum of donor age and recipient graft survival time. In parallel, we analyzed the cumulative liver age in a historical control cohort of recipients who received unperfused DBD livers at the University Hospital Zurich between 1986 and 2022.

Both cohorts, the HOPE-REAL DBD cohort and the unperfused DBD Zurich cohort, were compared in terms of liver age using propensity score matching, adjusting for donor age, recipient age, recipient MELD score, cold ischemia time, HCC diagnosis, and retransplantation.

### Statistical Analysis

Metric parameters were compared using the Mann-Whitney *U* test, whereas categorical variables were analyzed using χ^2^ test. Kaplan-Meier survival analysis was performed to assess overall graft survival over time, with time defined as cumulative liver age, and comparisons were made using the log-rank test. To address potential confounders, propensity score matching was conducted using logistic regression, including the following variables: donor age, recipient age, recipient MELD score, cold ischemia time, and HCC diagnosis. Nearest-neighbor 1:1 matching was applied, ensuring that the standardized mean difference remained below 0.1 for all variables. In addition, multivariate Cox regression analysis was performed to identify independent predictors of graft failure. Propensity score matching was performed using R software (R Core Team, 2024, version 4.4.2, R Foundation for Statistical Computing, Vienna, Austria).

## RESULTS

The HOPE-REAL DBD cohort comprised 768 cases, with a nearly equal distribution between dual-HOPE (n =387, 50.4%) and HOPE (n=381, 49.6%), collectively referred to as the HOPE group. The unperfused control cohort included 863 DBD liver transplants (control group).

**FIGURE 1:**
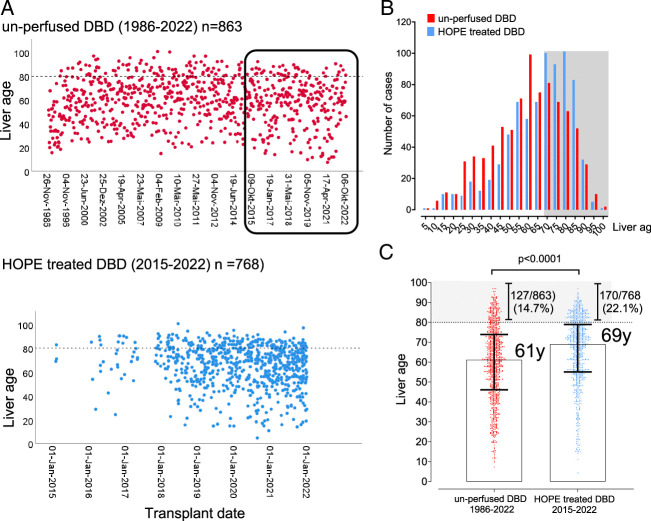
Distribution (scatter plot) of cumulative liver age throughout the observation periods in both cohorts. The number of cases during different observation periods, that is, 1986 to 2022 and 2015 to 2022 (A), and by liver age (B) is shown for the HOPE group and the control group, along with the median cumulative liver age (C) for each group.

**FIGURE 2:**
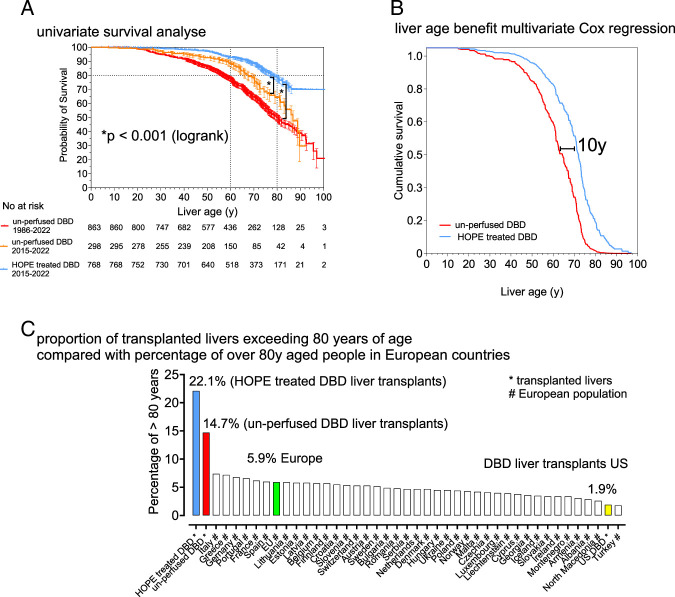
Univariate (A) and multivariate (B) analysis for cumulative liver age between the control group and the HOPE group. C, Comparison of the proportion of transplanted livers with donor age older than 80 years to the proportion of the aged European population (>80 years). (Source: Statistica 2020, URL: https://www.statista.com/statistics/1235624/share-of-elderly-population-in-europe-by-country/).

The median donor age in the HOPE group was 67 years (53–78) compared with 53 years [interquartile range (IQR): 37–65] in the control group (*P*<0.001). Recipient age was also significant higher in the HOPE group (59 vs 54 years, *P*<0.001), whereas cold ischemia time was 1.5 hours shorter (384 vs 480 minutes, *P*<0.001). Livers in the HOPE group were frequently transplanted into HCC recipients (45.6%), resulting in lower MELD scores (median: 14, IQR: 10–21). In contrast, the control group had fewer HCC cases (17.4%) and a higher MELD score (median: 21, IQR: 11–32) (Table 1). The distribution of cumulative liver age remained relatively stable throughout the observation period in both cohorts (Fig. 1). However, post-transplant outcomes were significantly better in the HOPE group (log-rank *P*<0.001, Fig. 2A), despite higher donor and recipient ages and a greater proportion of tumor cases, which resulted in a significantly higher cumulative liver age in the HOPE group (69 vs 61 years, *P*<0.001, Table 2). Notably, the liver life span benefit was independent of the study period, as cumulative liver age remained lower in the control group of unperfused DBD livers even in the most recent period (2015–2022) (Fig. 2A).

**TABLE 1:** Descriptive Characteristics of the HOPE-REAL DBD Cohort (HOPE Group), Compared With an Independent Cohort of Unperfused DBD Livers (Control Group)

	HOPE group 2012–2022 (n=768)	Control group 1986–2022 (n=863)	*P* *
Donor age (yr) (median/IQR)	67 (53–76)	53 (37–65)	<0.001
Graft cold ischemia time (CIT) (min) (median/IQR)	384 (267–480)	480 (381–595)	<0.001
Recipient age (yr) (median/IQR)	59 (52–65)	54 (45–61)	<0.001
Recipient lab MELD (median/IQR)	14 (10–21)	21 (11–32)	<0.001
Retransplantation rate (secondary or tertiary transplants, n (%)	38/768 (4.9)	75/860 (8.7)	0.003
HCC, n (%)	350/768 (45.6)	150/860 (17.4)	<0.001
Follow-up	2.4 yrs (1.4–3.7)	6.4 yrs (1.7–13.6y)	<0.001

* Untreated DBD (control) versus HOPE-treated DBD, Mann-Whitney *U* test.

**TABLE 2:** Cumulative Liver Age in the HOPE Group, Control Group, and in the United States (Data From the UNOS Registry)

Cumulative liver age	HOPE group 2012–2022 (n=768)	Control group 1986–2022 (n=863)	United States (UNOS Data) 1988–2021 (n=175,343)	*P* ^*^
Median/IQR	69 yrs (55–78.9)	61 yrs (46.1–73.9)	43 yrs (29.2–57.8)	<0.001^†^
>70 yrs, n (%)	372/768 (48.4)	261/863 (30.2)	14,001/175,343 (8.0)	<0.001^‡^
>80 yrs, n (%)	170/768 (22.1)	127/863 (14.7)	3462/175,343 (1.9)	0.001^‡^
>90 yrs, n (%)	20/768 (2.6)	25/863 (2.9)	513/175,343 (0.3)	0.764^‡^

^*^ HOPE group versus control group.

^†^ Mann-Whitney *U* test.

^‡^ Fisher exact test.

**FIGURE 3:**
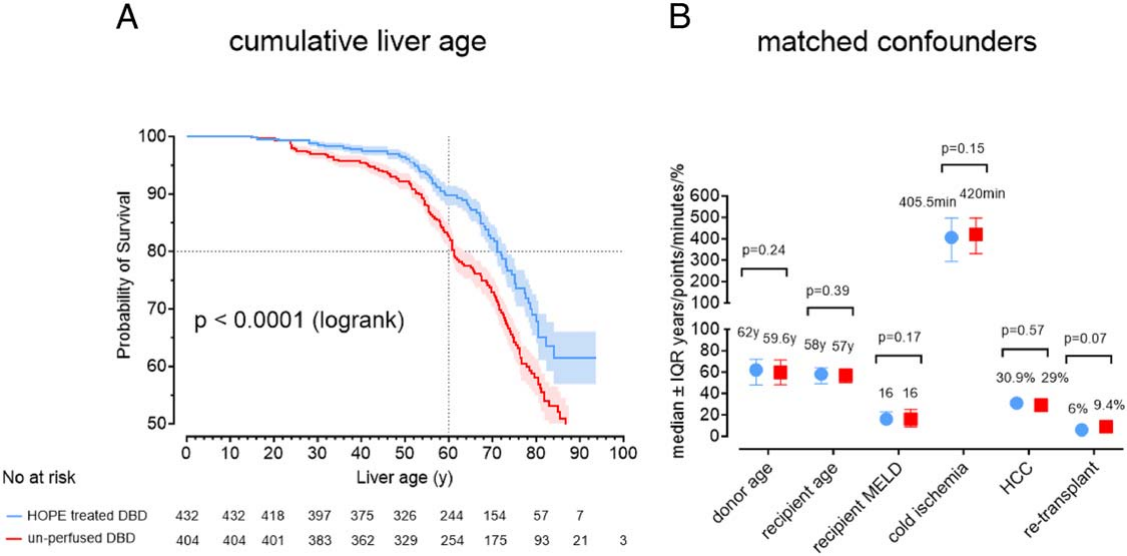
Cumulative liver age in relation to survival probability after propensity score matching in the HOPE group versus the control group (A), with key confounders adjusted in both cohorts (B).

Consistently, the number of livers reaching cumulative liver age of more than 70 years and more than 80 years in the HOPE group was 372/768 (48.4%) and 170/768 (22.1%), respectively, compared with 261/863 (30.2%) and 127/863 (14.7%) in the control group (*P*<0.001) (Table 2), both of which are considerably higher than those recently observed in the United States^11^ (Table 2). A separate analysis of liver transplants using donor livers older than 70 years or older than 80 years, revealed persisting and significant survival advantages for the HOPE group (Supplemental Figure 1, Supplemental Digital Content 1, http://links.lww.com/SLA/F580). Multivariate analysis identified HOPE treatment, along with donor age and primary transplantation, as a strong (*P*<0.001) and independent predictor of cumulative liver age (Supplemental Table 1, Supplemental Digital Content 1, http://links.lww.com/SLA/F580, Figure 2B). Furthermore, propensity score matching, adjusting for key confounders, confirmed significantly higher cumulative liver age in the HOPE-treated cohort (n=432) compared with a matched control group of unperfused DBD livers (n=404) (*P*<0.001, Fig. 3).

## DISCUSSION

In this study, we observed for the first time in a large cohort of HOPE-treated DBD livers that cumulative liver age was higher compared with a non-perfused control group. This suggests that HOPE treatment before implantation may influence aging processes, in addition to its known benefits for ischemia-reperfusion injury.^12^ Supporting this hypothesis, we found that the proportion of HOPE-treated livers reaching a cumulative liver age of over 80 years (22%) after transplantation was ~11 times higher than in a published US liver transplant cohort (1.9%; Table 2), and more than 3 times higher than in the general European population (5.9%; Fig. 2C).

The liver is the largest abdominal organ, known for its unique metabolic and regenerative capacities, and has been immortalized in numerous legends and poems. For example, in Greek mythology, the injured liver of Prometheus grows back every day for an unlimited time,^13^ and the liver is believed to be the seat of the human soul. Although it remains unclear how long a liver can live, the aging process of the liver seems to differ from other organs. Hepatocytes, for instance, contain an extraordinarily high number of 1000 to 2000 mitochondria and are known for their remarkable ability to regenerate throughout life, with an average hepatocyte age of <3 years.^14^ However, aging is an inevitable biological process that leads to a decline in organ and tissue function, contributing to various diseases, such as neurodegenerative, cardiovascular, metabolic, immune-mediated, and cancer-related conditions. Anti-aging efforts have been a longstanding topic, involving numerous procedures and treatments throughout history. Among these, organ transplantation, introduced in the last century, has become increasingly successful for treating a wide range of medical indications.^15^ Despite its success, organs transplantation is a complex procedure with several significant challenges, such as donor resuscitation, donor death, organ procurement, implantation, and post-implantation treatment, including immunosuppressive therapy. Surprisingly, recent data from the United States suggest that transplanted livers have life spans similar to the average of the general population,^11^ implying that older donor livers may not necessarily have a shorter survival. However, how old a liver can get after “changing its house” remains insufficiently explored.

Mitochondrial function has long been associated with aging, and mitochondrial quality control systems are thought to play a key role in maintaining low and steady level of mitochondrial oxidative stress.^16^ However, aging leads to disruption of these control processes, resulting in decreased efficiency of oxidative phosphorylation. This in turn affects ATP production, impacts on AMPK signaling, and increases the release of reactive oxygen species,^1^ which cause oxidative damage to mitochondrial DNA, proteins, and lipids. Disrupted mitochondrial quality control is therefore considered a critical contributor in the pathogenesis of many diseases^17^ and in the aging process itself.^18^

HOPE is known to enhance mitochondrial function in liver cells by metabolizing accumulated succinate and NADH.^10^ Correspondingly, HOPE-treated livers show improved mitochondrial complex I-IV activities, along with fully replenished cellular ATP pools.^10,19^ On the basis of this “mito-repair” effect, we postulate that HOPE treatment improves mitochondrial quality control in transplanted livers. Mechanistically, improved complex I function in HOPE-treated livers results in higher cellular NAD+ levels,^19^ as it reduces the release of complex I-bound flavin mononucleotide (FMN) (Supplemental Figure 2, Supplemental Digital Content 1, http://links.lww.com/SLA/F580). Reloaded cellular NAD+ levels have been the focus of several age-related studies,^20^ as NAD+ decreases with age, and supplementation with NAD precursors may prevent or slow the progression of various age-related diseases. Furthermore, graft injury during implantation has been shown to be significantly lower in HOPE-treated livers compared with unperfused livers,^21^ resulting in numerous clinically relevant benefits in the post-transplant period, including improved liver function, reduced complication rates, and better survival outcomes.^22,23^

An effect of ex situ liver perfusion on complex, multifactorial cellular processes is both interesting and novel, offering potential treatment options for injured organs. For instance, recent research suggests that machine perfusion of organs may potentially have anti-cancer effects,^24^ in addition to immune-modulatory effects,^25,26^ both of which require further investigation. However, the anti-aging effects of liver machine perfusion have yet to be recognized, although they could play a significant role in the concept of ex situ liver regeneration.^27^

This study has limitations. Because of its retrospective observational design, there are differences in several parameters between the compared groups, and varying follow-up periods. To minimize bias, we conducted a multivariate analysis adjusting for key confounders, and repeated comparison of liver age in 2 subgroups, both supporting the advantages of HOPE-perfused livers grafts. In addition, a propensity score match analysis confirmed significantly higher cumulative liver age in the HOPE-treated cohort compared with the unperfused control group (Fig. 3).

In conclusion, HOPE treatment may help mitigate the risks associated with transplanting livers from elderly DBD donors, enabling excellent long-term survival in an ageing recipient population. These findings warrant further investigation and support the use of HOPE for older donor livers, which could improve utilization rates and further expand the donor pool.

## Supplementary Material

## DISCUSSANTS

### José M. Ramia Angel (Alicante, Spain)

I would like to thank the ESA for the privilege of being the first discussant of this paper, and the authors for this interesting study.

I have an epidemiological question and 2 philosophical ones:

First, to begin with the epidemiological question, in your manuscript, it would be good to define what “elderly” means. When I was a resident, the term typically referred to individuals over 65, but today it often implies those over 80. For the clinical relevance of your study, I believe it’s important that all liver transplantation teams work from a consistent definition.

Second, moving to the philosophical questions, do you think that results from HOPE are extrapolable to other liver machine perfusion? In other words, do you think that any type of machine perfusion works in a different histological way?

Finally, if your findings suggest improved outcomes with elderly donors, why not apply this approach to all donors? Have you tested its efficacy across different donor groups?

### Response From Janina Eden (Groningen, The Netherlands)

Thank you very much for your thoughtful questions. The definition of “elderly” donor livers remains challenging, as liver utilization practices vary significantly between countries. For example, in the United States, the median donor age is around 40 years, whereas in Italy, a substantial number of livers from octogenarian donors are successfully used with excellent outcomes. Personally, I believe the term “elderly liver” should be reserved for livers from donors over the age of 80.

Second, we believe that the effects of HOPE are based on a unique mitochondrial reprogramming effect, driven by the metabolization of oxygen under cold conditions, which is not the case in other machine perfusion techniques. Future research is needed to understand potential anti-aging effects in normothermic liver perfusion strategies.

Third, we conducted separate analyses across different age groups and observed a similar effect to that seen in the overall cohort (Supplemental Figures, Supplemental Digital Content 1, http://links.lww.com/SLA/F580).

### Chase J Wehrle (Cleveland, Ohio, USA)

Congratulations, this is amazing and very exciting work. Yesterday, we talked about how liver aging is likely different. If cumulative liver age is the life of the liver before and after, your results are quite striking because the liver gets older in the transplanted population than the normal population, which seems to be different in the United States. One difference is hypothermic perfusion, which you point out; another difference is that we eat too much McDonald’s and drink too much bourbon in the United States, which is inherently bad. So, what do you think accounts for the difference between Europe and the United States in this context? Is there an additional aging-related factor in the liver that I may be overlooking, or do you believe the differences are due to other, more obvious factors?

### Response From Janina Eden (Groningen, The Netherlands)

I think that this is hard to answer because the United States and Europe are not really comparable. We utilize much older donors in Europe compared with the United States. This is a totally different setting.

### Comment From Philipp Dutkowski (Basel, Switzerland)

I just wanted to add a short comment. The pressure to use older livers, as well as DCD livers, is much higher in countries with longer waiting times. Therefore, the observed differences in donor age between Europe and the United States are most likely due to significant variations in organ availability across countries. For instance, the average waiting time for a liver transplant is ~18 months in Italy, 10 months in Switzerland, 4 months in the United States, and just 1 month in Spain. As a result, liver grafts in several European countries tend to carry a higher perceived risk compared with those in the United States.

### Christiane Bruns (Cologne, Germany)

Thank you very much for this question as it is in line with what I want to ask you. You mentioned mitochondrial damage and its influence on the HOPE group. Have you measured any specific markers of mitochondrial injury using liquid biopsies? For example, in the case of the 100-year-old liver that was retrieved for transplantation, did you assess any mitochondrial changes over time to evaluate whether HOPE helped rejuvenate mitochondrial activity in that graft?

### Response From Janina Eden (Groningen, The Netherlands)

No, we did not check this for these very old livers. This is actually the first report on the effect of machine liver perfusion on liver age. What we do know is that there is a protective effect of HOPE on mitochondria, and mitochondria play a crucial role in cellular aging. More data are needed to investigate the mechanistical background.
