# Supplementary material for: Life Expectancy of Transplanted Livers: HOPE Against Aging?
Source: Ann Surg. 2025 Aug 7;282(5):765–71. doi: 10.1097/SLA.0000000000006883 (PMC12513047; doi:10.1097/SLA.0000000000006883)
Supplement: Supplementary file 1 [file sla-282-765-s001.docx]

**Life expectancy of transplanted livers – HOPE against aging?**

*Janina Eden, MD^1,2,3^ , Philip C Müller, MD^3^, Christoph Kuemmerli, MD^3^, Noa Aegerter, MD^3^, Isabel MA Brüggenwirth, MD^1,2^, Gabriela Berlakovich, MD^4^, Bettina M Buchholz, MD^5^, Florin Botea, MD^6^, Stefania Camagn, MDi^7^, Matteo Cescon, MD^8^,* *Umberto Cillo, MD PhD^9^, Fabio Colli, MD^10^, Luciano G. De Carlis, MD^11^, Riccardo De Carlis, MD^11^, Fabrizio Di Benedetto, MD^12^, Jule Dingfelder, MD^4^, Dulce Diogo, MD^13^, Daniele Dondossola, MD^14^, Moritz Drefs, MD^15^, Jiri Fronek, MD PhD^16^, Giuliana Germinario, MD^8^, Enrico Gringeri, MD^9^, Christiano Guidetti, MD^12^, Georg Györi, MD^4^, Matej Kocik, MD^17^, Efrayim H. Küçükerbil, MD^17^, Dionysios Koliogiannis, MD^15^, Georg Lurje, MD^18^, Paolo Magistri, MD^12^, Diethard Monbaliu, MD PhD^19^, Mostafa el Moumni, MD^20^, Beat Müller, MD^3^, Damiano Patrono, MD^10^, Wojciech G Polak, MD^17^, Robert J Porte, MD PhD^2,17^, Matteo Ravaioli, MD^8^, Michel Rayar, MD^21^, Renato Romagnoli, MD PhD^10^, Gustaf Sörensen, MD^22^, Deniz Uluk, MD^18^, Pierre A Clavien, MD PhD^23^, Vincent E de Meijer, MD PhD^1,2#^* , *Philipp Dutkowski, MD^3,23#^*

*^#^ shared senior authorship.*

**Affiliations:**

1. Department of Surgery, Section of HPB Surgery and Liver Transplantation, University of Groningen and University Medical Center Groningen, Groningen, the Netherlands
2. UMCG Comprehensive Transplant Center, Groningen, the Netherlands
3. Department of Visceral Surgery, University of Basel, Clarunis, Basel, Switzerland
4. Division of Transplantation, Medical University of Vienna, Vienna, Austria
5. Department of Visceral Transplantation, University Medical Center Hamburg-Eppendorf, Hamburg, Germany
6. Fundeni Clinical Institute, Center of General Surgery and Liver Transplantation; "Titu Maiorescu" University, Bucharest
7. Department of Organ Failure and Transplantation, ASST Papa Giovanni XXIII, Bergamo, Italy
8. Department of General Surgery and Transplantation, IRCCS, Azienda Ospedaliero-Universitaria of Bologna, University of Bologna, Bologna, Italy
9. Chirurgia Generale 2, Hepato-Biliary-Pancreatic Unit and Liver Transplant Center, Padova University Hospital, Padova, Italy
10. General Surgery 2U, Liver Transplant Centre, Azienda Ospedaliero Universitaria Città della Salute e della Scienza di Torino, Turin, Italy
11. Department of General Surgery and Transplantation, ASST Grande Ospedale Metropolitano Niguarda, Milan, Italy
12. Hepato-Pancreato-Biliary Surgery and Liver Transplantation Unit, University of Modena and Reggio Emilia, Modena, Italy
13. Adult Liver Transplantation Unit, Department of Surgery and Gastroenterology, Coimbra Hospital and University Center, Coimbra, Portugal
14. General and Liver Transplant Surgery Unit, Fondazione IRCCS Ca’ Granda Ospedale Maggiore Policlinico, and Department of Pathophysiology and Transplantation Università degli Studi di Milano, Milan, Italy
15. Department of Transplant Surgery, University of Munich Grosshadern, Gemany
16. Transplant Surgery Department, Institute for Clinical and Experimental Medicine (IKEM), Prague, Czech Republic
17. Erasmus MC Transplant Institute, University Medical Center Rotterdam, Division of HPB and Transplant Surgery, Rotterdam, the Netherlands
18. Department of Surgery, Universitätsklinikum Heidelberg, Heidelberg, Germany
19. Department of Abdominal Transplantation, Leuven Transplant Center, University Hospitals Leuven, Leuven, Belgium
20. Department of Surgery, Section of Epidemiology and Statistics, University of Groningen and University Medical Center Groningen, Groningen, the Netherlands
21. CHU Rennes, Service de Chirurgie Hépatobiliaire et Digestive, Rennes, France
22. Transplant Institute, Sahlgrenska University Hospital, Gothenburg, Sweden
23. Swiss HPB and Transplant Center, Department of Visceral Surgery and Transplantation, University Hospital Zurich, Zurich, Switzerland

^All authors declare nothing to disclose.^

Abstract: 223 (<= 250 words)

Miniabstract: 29

Manuscript: 2020 (<= 2600 Words)

3 Figures, 2 Tables, Supplemental Figure 1&2, Supplemental Table 1

Correspondence:

P. Dutkowski

Department of Visceral Surgery,

University of Basel, Clarunis

Basel, Switzerland

[philipp.dutkowski@usb.ch](mailto:philipp.dutkowski@usb.ch)

**Supplemental Figure 1:** Analysis of key risk factors (A, C) and survival outcomes (B, D) in subgroups of donors aged >70 years and >80 years. A comparison of the HOPE group and control group is shown.

**Supplemental Figure 2:** The relationship between mitochondrial complex I injury and cellular aging (A), and the postulated mitochondrial repair process by HOPE treatment (B).

**Supplemental Table 1:** Multivariate cox regression analysis on cumulative liver age with categorized parameters in the HOPE group

| Hazard ratios | Variable | Estimate | 95% CI  (profile likelihood) | p value |
| --- | --- | --- | --- | --- |
| exp(β1) | **primary transplant (y/n)** | 4.159 | 2.729 to 6.169 | **< 0.001** |
| exp(β2) | HCC (y/n) | 1.070 | 0.750 to 1.524 | 0.707 |
| exp(β3) | **HOPE treatment (y/n)** | 0.432 | 0.319 to 0.585 | **<0.001** |
| exp(β4) | **donor age <> 59y *** | 0.071 | 0.045 to 0.109 | **<0.001** |
| exp(β5) | recipient age <> 57y * | 1.230 | 0.926 to 1.637 | 0.155 |
| exp(β6) | MELD <> 16 * | 1.198 | 0.856 to 1.683 | 0.296 |
| exp(β7) | CIT <> 424min * | 0.846 | 0.637 to 1.121 | 0.247 |

* variables categorized by median
